# The pattern and use of Twitter among dental schools in Saudi Arabia

**DOI:** 10.1371/journal.pone.0272628

**Published:** 2022-09-08

**Authors:** Khalifa S. Al-Khalifa, Fatimah N. AlMuhammadi, Noor Y. AlOraifi, Elaf A. Alkuwaiti, Banan A. Aladinan, Nada M. Alzahrani, Sarah A. Khusheim, Mahmoud H. Al-Johani

**Affiliations:** 1 Department of Preventive Dental Sciences, College of Dentistry, Imam Abdulrahman Bin Faisal University, Dammam, Saudi Arabia; 2 Dental Internship Program, College of Dentistry, Imam Abdulrahman Bin Faisal University, Dammam, Saudi Arabia; 3 Restorative Dental Department, East Jeddah Hospital, Ministry of Health, Jeddah, Saudi Arabia; The University of Adelaide, AUSTRALIA

## Abstract

**Objective:**

Twitter as a social media platform has revolutionized the way we interact with others and receive information. The presence of dental schools in Twitter facilitates the engagement of students, educators, dental professionals, and the community. Given the explosive popularity of Twitter as a social media platform and its potential use in the areas of education and branding, the questions of why and how dental schools use these services warrant comprehensive research. Thus, the aim of this study was to analyze the pattern and use of Twitter as a social media platform for dental schools in Saudi Arabia.

**Methods:**

The tweets were extracted within the timeframe from July 15, 2019, to July 15, 2020. The Twitter data collected included: full text content, the count of retweets, quotes, replies and likes. Extracted tweets were categorized into five main themes: news and announcement, dental professional communication, general communication, oral health education, and promoting participation. Tweets in each main theme were further categorized according to the dental schools’ academic roles namely; education, research and community service. In addition, tweets were classified according to originality of the tweet, language used, nature of the tweet and the use of hashtags and mentions. Descriptive analysis presented in the form of frequency tables with percentages and mean (SD) as well as graphical presentation of the pattern and use of Twitter for Saudi dental schools in the form of bar, pie and line charts. Categorical data were analyzed using chi square test, while continuous data were analyzed using ANOVA. Statistical significance was set at p ≤ 0.05.

**Results:**

A total of 15 Saudi dental schools with Twitter accounts were included in the analysis. King Saud University (KSU) had the largest number of followers with 17,200. Within the time frame of this study, a total of 1,889 original tweets from dental schools were found. Imam Abdulrahman Bin Faisal University (IAU) had the highest number of posted tweets (n = 647, 34.3%). The distribution of tweets was highest in September 2019 (n = 239) and lowest in July 2020 (n = 22). Majority of the tweets (81.9%) belonged to five out of the 15 dental schools. News and announcements were the most tweeted thematic subject with 1,034 tweets (55%). While community service was the most tweeted academic role with 803 tweets (42%). The top five active dental schools’ performance for both thematic and academic role classifications were significantly different based on the chi square test (p < 0.001).

**Conclusion:**

This study highlights the importance of Twitter as a social media platform, in dental education especially when it comes to presence and branding for dental schools. Twitter is a helpful platform to expose dental schools to the community, this can be seen by their academic achievements as well as their active role with community service.

## Introduction

Twitter as a social media platform has revolutionized the way we live our lives, interact with others, and receive information [1,2]. Nowadays, millions of users are present on Twitter and the numbers are rising exponentially [2]. Currently in Saudi Arabia, more than half of the population use social media, accounting to 18.3 million and Twitter is among the most used platforms with nearly 9 million users [3]. Twitter has become a great opportunity to expand the educational and academic networks where medical professionals and students can communicate easily and promote shared interests [4,5]. Twitter offers effortless accessibility to health information and convenient knowledge exchange; it provides rich clinical educational content through threaded tweets and spaces [4,6]. Additionally, medical professionals are utilizing Twitter to share their published research papers, and according to multiple studies, those articles that are shared on Twitter are more likely to be cited [7,8]. Twitter is also considered as a critical tool for patient education [4].

Dental schools have utilized Twitter for a variety of purposes, including dental education promotion, communication with students and faculty, interaction with the community, sharing information, updates, and announcing activities [5,9]. The presence of dental schools in social media and Twitter, in specific, facilitates the engagement of students, educators, dental professionals, and the community thus reaching a broader group of people [10,11]. Dental schools’ Twitter accounts work as channels to promote oral health and raise dental awareness of the community [11]. Moreover, they provide high school graduates, who depend on social media, the essential information about schools’ life and academia [10]. As for dental students, it is helpful to bring them together and create a sense of affiliation towards their dental schools [10]. Furthermore, in a recent study, 83% of patients seem to have an interest in dental health providers who are active in social media [12]. Thus, it is necessary to highlight the importance of Twitter among dental schools, as it is considered a powerful tool that manages to bring both the dental professionals and the community together [2].

There is no literature about dental schools’ presence on twitter, what is their role, type of messages being delivered, quality of messages and how could it be improved. Given the explosive popularity and growth of Twitter as a social media platform and its potential use in the areas of education as well as marketing and branding, the questions of why and how dental schools use these services warrant comprehensive research. The use of Twitter to support the vision and mission of dental schools in achieving their goals whether with dental students, faculty members or patients’ needs to be addressed. Thus, the aim of this study was to analyze the pattern and use of Twitter as a social media platform for dental schools in Saudi Arabia.

## Materials and methods

This cross-sectional, observational study was carried during a period of one year from July 2019 to July 2020. This study used publicly available data that did not involve human subjects; thus, it did not require an institutional review board approval from Imam Abdulrahman Bin Faisal University, Dammam, Saudi Arabia.

### Search strategy

The number of licensed Saudi dental schools was officially obtained from the Saudi Arabian Ministry of Education [13]. This was followed by including only official Twitter accounts of these dental schools. Tweets from supporting accounts (e.g.: Main university accounts, clubs, or hospital accounts) were excluded. The tweets were extracted within the assigned timeframe. Twitter data collected included: the dental schools Twitter ID, full text content, and the count of retweets, quotes, replies and likes. The search was done manually where all tweets were collected by four investigators (SK, BA, NZ and EK) which was then entered in an EXCEL spreadsheet for further analysis.

### Coding of tweets

Extracted tweets were categorized into five main themes: news and announcement, dental professional communication, general communication, oral health education, and promoting participation. Furthermore, the tweets in each main theme were further categorized into three sub-categories according to the dental schools’ academic roles namely; education, research and community service. Tweets of irrelevant content to the scope of the study were excluded. The examples for the main themes with sub-categories can be seen in Table 1. The thematic analysis of tweets was performed in a similar manner to previous studies [14,15].

**Table 1:** Examples for thematic classification and academic role of dental schools’ tweets.

Thematic classification	Academic role	Example
News and Announcement	Education	Announcement of 1^st^ research seminar in the internship program.
	Research	Announcing first place winner in observational studies.
	Community service	Announcement of the field visits program in mobile clinics for the current academic year.
Dental Professional communication	Education	Educational videos links for teeth preps
	Research	Information about virtual poster session.
	Community service	Announcement of live webinar by IADR Saudi Division.
General communication	Education	Welcoming message for students who have been accepted in College of Dentistry.
	Research	A thank you message for the organizing committee of Annual IAU Dental Research Day.
	Community service	Listing some facts about COVID-19 disease.
Oral health Education	Education	None
	Research	None
	Community service	Listing some truth and myths about oral and dental health.
Promoting Participation	Education	A Twitter survey to share expectation about best batch participating in students’ activities in college of dentistry.
	Research	A Twitter survey to share expectation about best batch participating in scientific research in college of dentistry.
	Community service	A Twitter survey to share expectation about best batch participating in community service in college of dentistry.

Furthermore, the tweet content was classified according to originality of the tweet whether it was original or a retweet. In addition, tweets were classified based on the language used either Arabic, English or both. The nature of the tweet content was classified to either text, media or both. Another form of classification of tweets was to assess hashtags (#), where they were counted per tweet and they were classified into dental school’s own hashtag, common dental related, common subject related, trending unrelated or combination of hashtags. Tweet content was assessed in terms of the number of mentions (@) and further this was classified into organizational mention, personal mention, or both.

The coding was performed by two trained and calibrated investigators (FM and NO). Both coders conducted a pilot by coding 100 random posts. A weighted Kappa was utilized to test the reliability. Both investigators classified another 200 randomly selected tweets and were reclassified one week after the first coding. The weighted Kappa was 0.836 for inter-agreement and 0.865 (FM) and 0.889 (NO) for intra-agreement with an overall excellent reliability. Any conflict in coding was resolved by a gold standard examiner (KK) via discussion to ensure full understanding of tweets categories and themes by both investigators.

### Data analysis

The data was entered in Microsoft Excel (2010) and transferred to IBM SPSS Statistics for Windows, version 22 (IBM Corp., Armonk, NY, USA) for statistical analysis.

Descriptive analysis was performed to present the overview of the findings. The results were presented in the form of frequency tables with percentages and mean (SD) as well as graphical presentation in the form of pie graphs. In addition, the pattern of Twitter use by Saudi dental schools over a ‘one year’ time period examined per month, as well as comparing the Twitter use of the top five Saudi dental schools were presented in the form of bar and line graphs, respectively. Categorical data were analyzed using chi square test, while continuous data were analyzed using ANOVA. Statistical significance was set at p ≤ 0.05.

## Results

A total of 15 Saudi dental schools with social media accounts on Twitter were included in the analysis. The School of Dentistry, Qassim University (Main Campus in Buraidah) was the first to establish a Twitter account in June 2009, and the School of Dentistry, King Faisal University (KFU) was the latest to join Twitter in August 2019. The School of Dentistry, King Saud University (KSU) had the largest number of followers with 17,200 followers, while KFU had the least number of followers, with only 117 followers. Within the time frame of this study, a total of 1,889 original tweets from dental schools were found. The School of Dentistry, Imam Abdulrahman Bin Faisal University (IAU) had the highest number of posted tweets (n = 647, 34.3%). While, The Schools of Dentistry in Dar Al-Uloom University and Al-Qassim University (Arrass Campus) both had the least number of posted tweets (n = 13, 0.7%) (Table 2).

**Table 2:** Twitter accounts information and number of tweets per dental school (July 2019- July 2020).

Dental College	Twitter (@Username)	Twitter Join Date	Number of followers	Number of tweets N = 1889 n (%)
Imam Abdulrahman Bin Faisal University (IAU)	IAUdent	September 2011	4463	647 (34.3)
King Saud University (KSU)	ksudental	September 2010	17200	298 (15.8)
King Abdulaziz University (KAU)	KAU_FD	Dec, 2014	2453	271 (14.3)
Umm Al-Qura University (UQU)	uqudent	June 2010	6280	180 (9.5)
Prince Sattam Bin Abdulaziz University (PSAU)	dentistry_psau	September 2014	866	152 (8)
Jazan University	Jazandental	October 2016	3000	109 (5.8)
King Saud bin Abdulaziz University for Health Sciences (KSAUH)	KSAU_HS_COD	June 2012	3118	45 (2.4)
Qassim University (Buraidah)	qudent	June 2009	2292	34 (1.8)
Najran University	dent_nu	April 2015	2088	30 (1.6)
Taibah University	taibah_dental	November 2014	2675	30 (1.6)
King Khalid University (KKU)	KKU_Dental	September 2012	3154	27 (1.4)
Jouf University	JU_dent	December 2011	2331	24 (1.3)
King Faisal University *(*KFU)	dentistrykfu	August 2019	117	16 (0.8)
Dar al uloom University	CDT_DAU	July 2017	199	13 (0.7)
Qassim University (Rass)	RassDentCollege	December 2018	234	13 (0.7)

**Fig 1:**
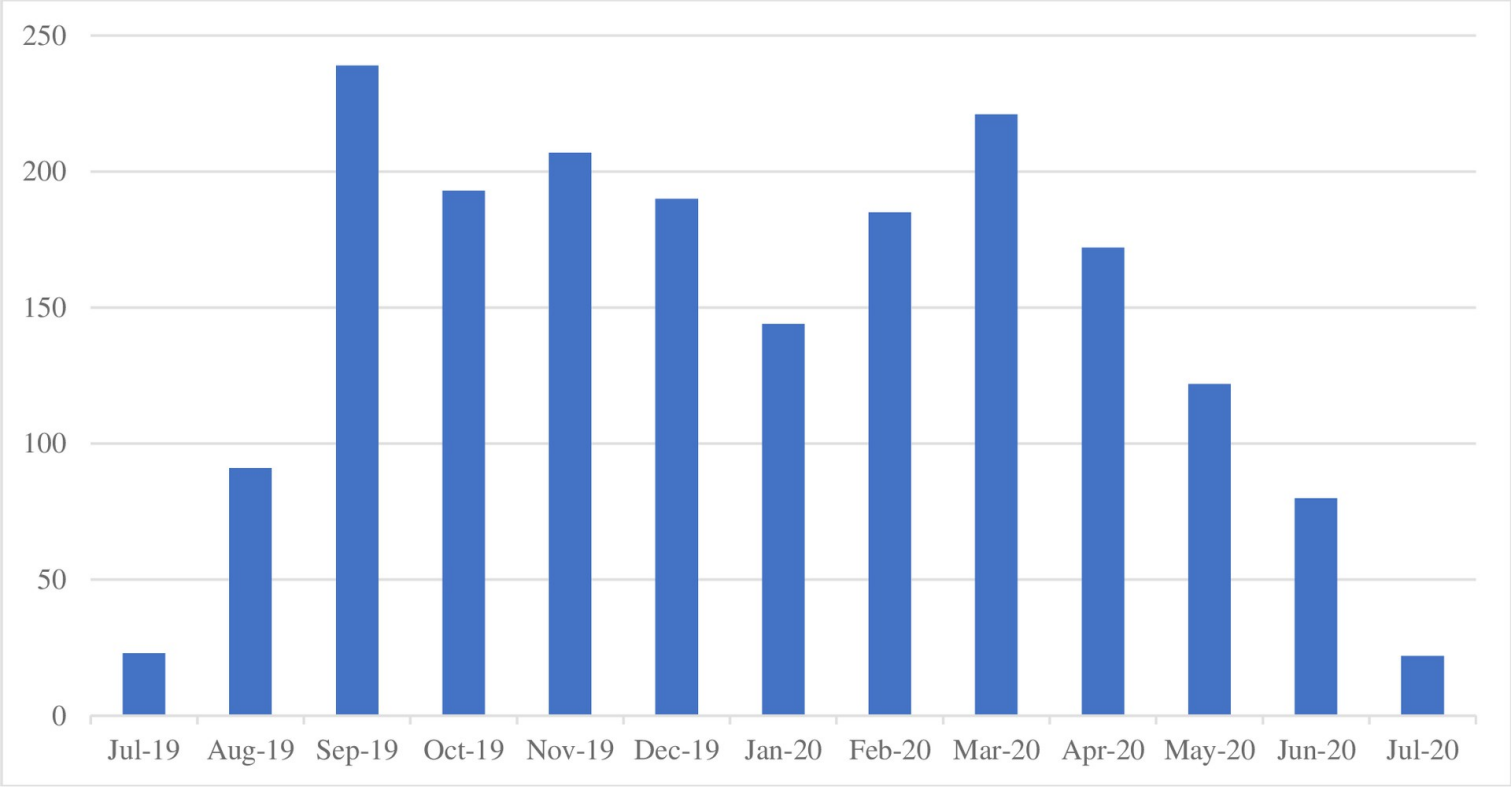
Distribution of Tweets per month (July 2019—July 2020).

The distribution of tweets per month from July 15, 2019, to July 15, 2020, is shown in Fig 1. The number of tweets were low (n = 23) in July 2019 but started to rise in the following months where it reached the highest in September 2019 (n = 239). The number of tweets dropped after September 2019 where it was the lowest in January 2020 (n = 144). The number of tweets increased again in March 2020 (n = 221) then started to drop until it reached 22 tweets in July 2020.

**Fig 2:**
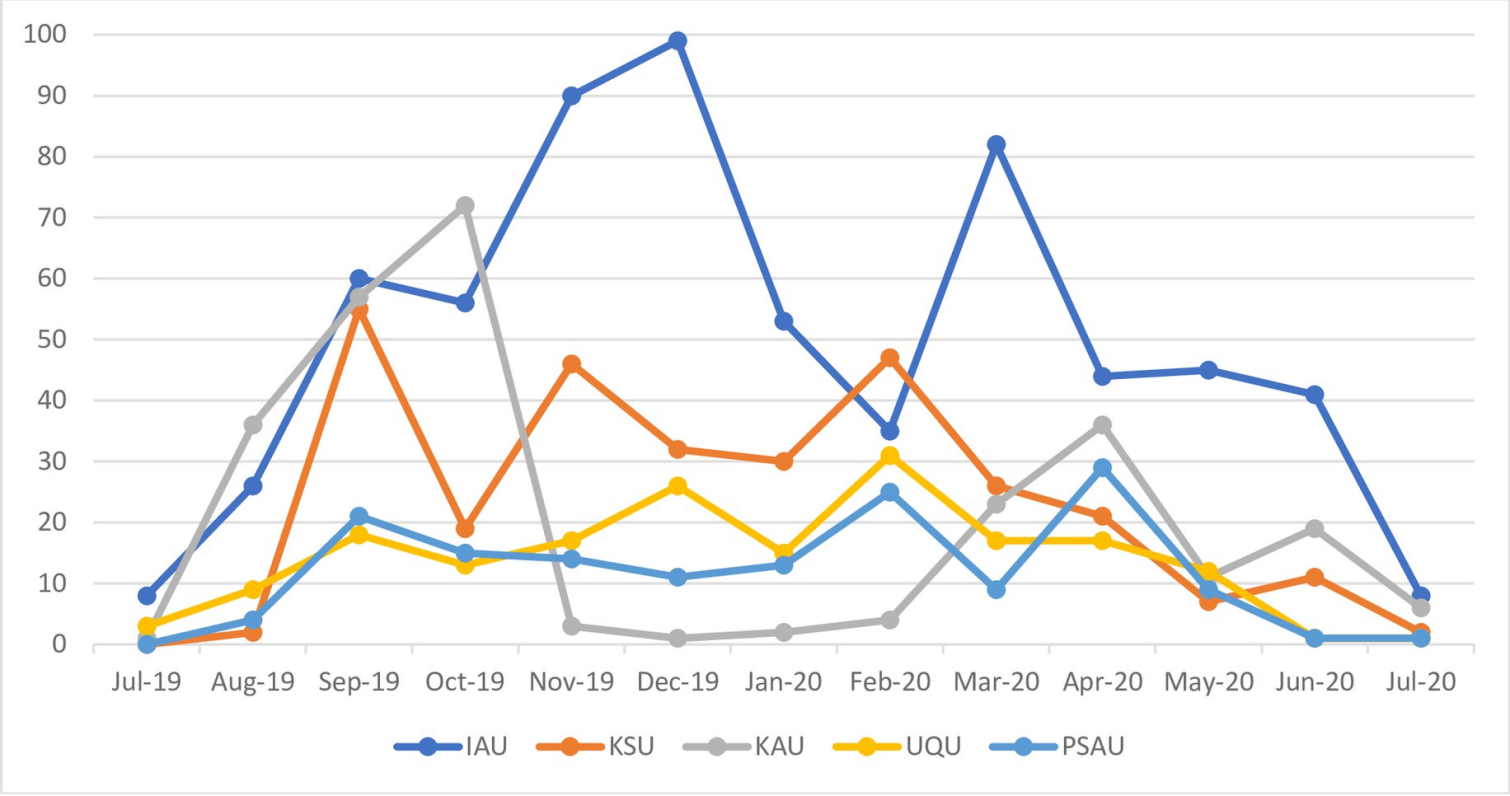
Time distribution of tweets for top five dental schools from July 2019 to July 2020. **IAU:** Imam Abdulrahman Bin Faisal University, **KSU:** King Saud University, **KAU:** King Abdulaziz University, **UQU:** Umm Al-Qura University, **PSAU:** Prince Sattam Bin Abdulaziz University.

It was noticed during data analysis that the majority of the tweets (81.9%) belonged to five dental schools: IAU (n = 647, 34.3%), KSU (n = 298,15.8%), King Abdulaziz University (KAU) (n = 271,14.3%), Umm Al-Qura University (UQU) (n = 180, 9.5%) and Prince Sattam Bin Abdulaziz University (PSAU) (n = 152, 8%). Furthermore, Fig 2 shows the time distribution of tweets for the top five dental schools from July 2019 to July 2020. It was noticed that the top five dental schools’ tweets started to increase from July 2019 through September 2019. Four out of the five top dental schools dropped in the number of tweets in October 2019 except for KAU where it reached its highest activity then it decreased in the following months. The number of tweets for IAU increased in November 2019 and reached its highest activity in December 2019. The month of January 2020 noticed a drop in the number of tweets for most of the top five dental schools. The tweets activity for the top five dental schools kept going up and down from February 2020 through May 2020 with the most noticeable increase in tweets for IAU in March 2020. The month of July 2020 exhibited the lowest tweets activity for the top five dental schools.

**Fig 3:**
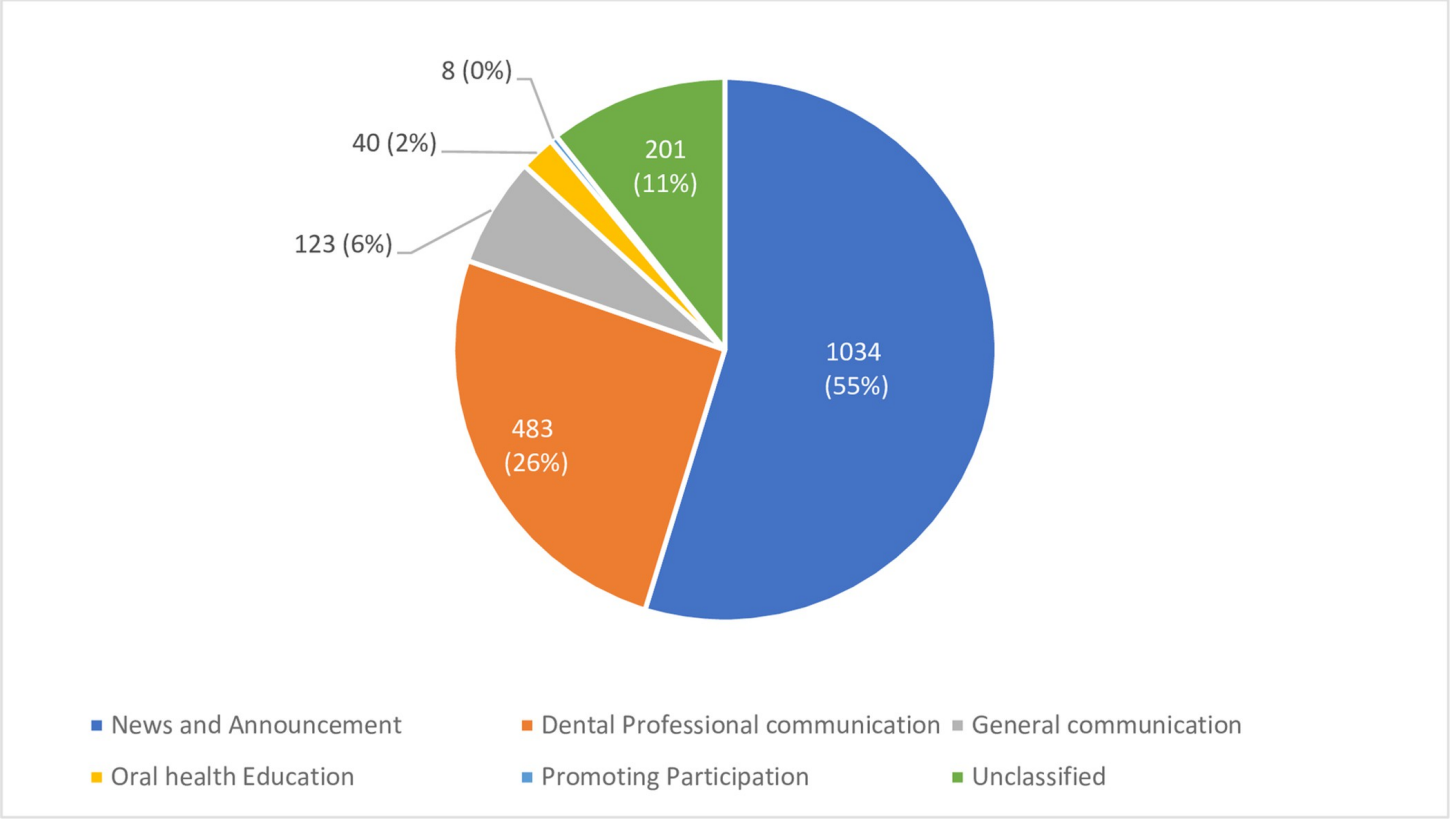
Thematic distribution of tweets for all dental schools.

**Fig 4:**
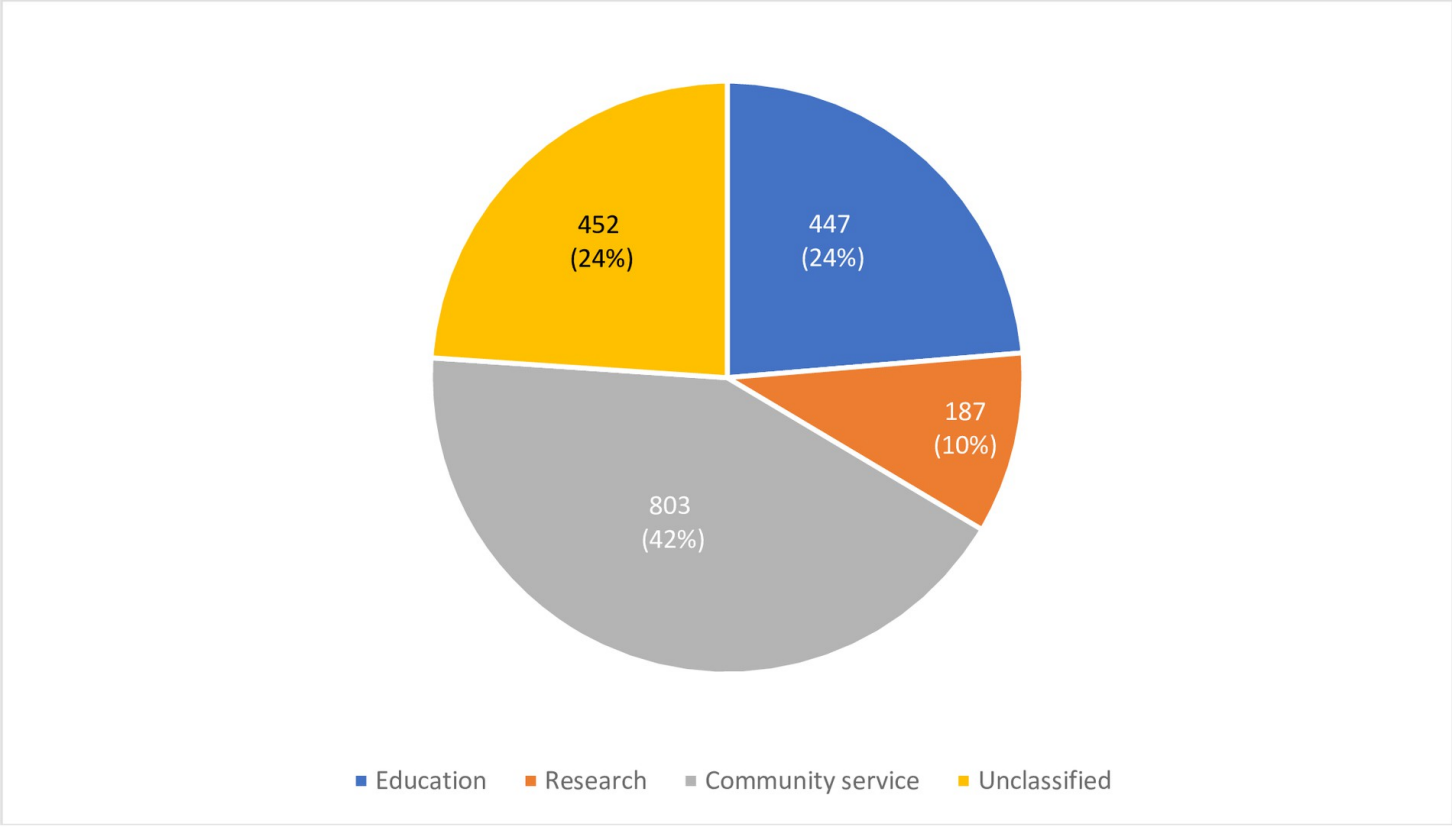
Distribution of tweets per academic role for all dental schools.

Fig 3 presents the tweets per thematic classification for all 15 dental schools. News and announcements were the most tweeted subject with 1,034 tweets (55%), followed by dental professional communication with 483 tweets (26%), whereas promoting participation was the least tweeted subject with only eight tweets. Similarly, Fig 4 displays the tweets per academic role classification for all 15 dental schools. Community Service was the most tweeted subject among all dental schools with 803 tweets (42%), followed by education with 447 tweets (24%) and research was the least tweeted subject with 187 tweets (10%).

In addition to the previously mentioned classifications (thematic and academic role), the tweets were further classified by language, content nature, and the type of hashtags and mentions. Table 3 describes the distribution of tweets in each classification and category among the top five active dental schools. Under thematic classification, news and announcements tweets were the highest in IAU (55.0%), KSU (53.7%), UQU (57.2%) and PSAU (55.9%). On the other hand, KAU was mostly focusing on dental professional communication (50.2%). As for the academic role, community service tweets were found to be the highest in both KAU and IAU, with and (62.4%) and (53.5%), respectively. While research was found to be the lowest tweeted subject among dental schools (Table 3). The top five active dental schools’ performance for both thematic and academic role classifications were significantly different based on the chi square test (p < 0.001).

**Table 3:** Distribution of tweets according to the study variables for the top five dental schools.

Variable		Dental College				
		IAU	KSU	KAU	UQU	PSAU
		n (%)				
**Thematic classification**	News and Announcement	356 (55.0)	160 (53.7)	83 (30.6)	103 (57.2)	85 (55.9)
	Dental Professional communication	208 (32.1)	58 (19.5)	136 (50.2)	11 (6.1)	38 (25.0)
	General communication	24 (3.7)	10 (3.4)	32 (11.8)	16 (9.0)	12 (7.9)
	Oral health Education	30 (4.6)	0 (0.0)	1 (0.4)	8 (4.4)	0 (0.0)
	Promoting Participation	5 (0.8)	0 (0.0)	1 (0.4)	1 (0.6)	0 (0.0)
	Unclassified	24 (3.7)	70 (23.5)	18 (6.6)	41 (22.8)	17 (11.2)
**Academic role**	Education	148 (23.0)	65 (21.8)	45 (16.6)	43 (24.0)	29 (19.1)
	Research	59 (9.1)	19 (6.4)	23 (8.5)	13 (7.2)	15 (9.9)
	Community Service	346 (53.5)	94 (31.5)	169 (62.4)	61 (34.0)	52 (34.2)
	Unclassified	94 (14.5)	120 (40.3)	34 (12.5)	63 (35.0)	56 (36.8)
**Language**	Arabic	585 (91.0)	238 (81.8)	117 (45.5)	179 (99.4)	133 (92.4)
	English	42 (6.5)	45 (15.5)	137 (53.3)	1 (0.6)	11 (7.6)
	Both	17 (2.6)	8 (2.7)	3 (1.2)	0 (0.0)	0 (0.0)
**Content Nature**	Text	36 (5.6)	46 (15.5)	34 (12.6)	52 (29.0)	3 (2.0)
	Media	23 (3.6)	154 (51.8)	40 (15.0)	1 (0.6)	91 (60.3)
	Both	588 (91.0)	97 (32.7)	195 (72.5)	127 (70.6)	57 (37.7)
**Hashtag Type**	Own Hashtag	79 (12.9)	10 (19.6)	37 (19.2)	21 (13.0)	2 (4.1)
	Common dental related	48 (7.8)	0 (0.0)	0 (0.0)	0 (0.0)	0 (0.0)
	Common subject related	19 (3.1)	40 (78.4)	31 (16.1)	8 (23.5)	45 (91.8)
	Trending unrelated	3 (0.5)	0 (0.0)	0 (0.0)	0 (0.0)	1 (2.0)
	Combination	464 (75.7)	1 (2.0)	125 (64.8)	5 (14.7)	1 (2.0)
**Mention Type**	Organizational mention	3 (43.0)	0 (0.0)	32 (59.3)	11 (64.7)	2 (66.7)
	Personal mention	2 (28.6)	0 (0.0)	6 (11.1)	4 (23.5)	1 (33.3)
	Both	2 (28.6)	0 (0.0)	16 (29.6)	2 (11.8)	0 (0.0)

**IAU:** Imam Abdulrahman Bin Faisal University, **KAU:** King Abdulaziz University, **KSU:** King Saud University, **UQU:** Umm Al-Qura University, **PSAU:** Prince Sattam Bin Abdulaziz University.

Furthermore, the top five dental schools mostly used Arabic language in their content, except for KAU, which preferred English language instead (53.3%). The most used content for the tweets was a combination of both text and media as seen in IAU (91.0%), KAU (72.5%) and UQU (70.6%). However, media was used alone more in PSAU (60.3%) and KSU (51.8%). In addition, most of the top five dental schools were either using a common subject related or combination type of hashtags. For instance, PSAU most commonly used a common subject related hashtag (91.8%), while IAU more likely used the combination type of hashtags (75.7%). For the mention type, organizational mentioning was the highest among IAU, KAU, UQU and PSAU (Table 3). The top five active dental schools’ performance for language, content nature, hashtag and mention type were all significantly different based on the chi square test (p < 0.001).

Table 4 shows the mean of each type of tweets engagement, where UQU scored the highest number of received retweets and likes with 10.7±13.4 and 26.4±19.7, respectively. Moreover, KSU scored the highest number of received comments (3.9±10.3), and KAU scored the highest number of used hashtags and mentions with 3.9±4.9 and 0.6±1.4, respectively. The top five active dental schools’ mean performance for the number of retweets, likes, comments, hashtags and mentions were all significantly different based on the ANOVA test (p < 0.001).

**Table 4:** Measures of tweets engagement for the top five dental schools.

Variable	Dental College				
	IAU	KSU	KAU	UQU	PSAU
	Mean ± SD				
**Number of Retweets**	6.7±10.4	6.4±13.4	6.9±9.3	10.7±13.4	5.5±4.4
**Number of Likes**	13.4±16.3	15.4±24.9	11.9±18.1	26.4±19.7	10.6±9.0
**Number of Comments**	1.2±4.2	3.9±10.3	1.0±4.5	2.9±7.9	1.9±7.3
**Number of Hashtags**	2.6±1.7	0.2±0.4	3.9±4.9	0.3±0.7	0.3±0.5
**Number of Mentions**	0.0±0.3	0.0±0.0	0.6±1.4	0.2±0.6	0.0±0.2

**IAU:** Imam Abdulrahman Bin Faisal University, **KSU:** King Saud University, **KAU:** King Abdulaziz University, **UQU:** Umm Al-Qura University, **PSAU:** Prince Sattam Bin Abdulaziz University.

Table 5 demonstrates the top retweeted, liked, and commented tweet’s topics among all 15 dental schools. IAU, KSU, and KKU had the highest retweeted, liked, and commented topics, respectively. All topics were classified as news and announcements under the thematic classification. As for the academic role, only education was noticed among the retweeted and liked tweets. The highest retweeted topic was about the graduation of IAU fourth female dental batch with 106 retweets. In addition, the highest liked topic was by KSU announcing the first doctorate thesis statement with 179 likes. While the highest commented topic included the death news of a dental student studying in KKU, with 121 comments.

**Table 5:** Top five retweeted, liked and commented topics for all dental schools.

**Top 5 Retweeted Topics**	**Thematic Classification**	**Academic** **Roles**	**Number of Retweets**
Online graduation ceremony of the fourth IAUdent female batch.	News and announcements	Education	106
First doctorate thesis presentation in pediatric dentistry program in the college of dentistry (COD) at KSU.		Education	92
Announcement: postponing midterm exams and all academic activities at IAU COD until further notice.		Education	92
Congratulating a faculty member for appointing her as the Dean of the College of Dentistry IAU.		Unclassified	88
Announcement: COD at KAU accreditation from CODA on its educational program.		Education	82
**Top 5 Liked Topics**	**Thematic Classification**	**Academic** **Roles**	**Number of Likes**
First doctorate thesis presentation in pediatric dentistry program in the COD at KSU.	News and announcements	Education	179
Congratulating a faculty member for appointing her as the Dean of the College of Dentistry at IAU.		Unclassified	177
COD at KAU congratulating a faculty member for her promotion to full professor.		Unclassified	138
Doctorate thesis presentation and acceptance in the COD at KSU.		Education	136
Announcement: COD at KAU got accreditation from CODA on its educational program.		Education	117
**Top 5 Commented Topics**	**Thematic Classification**	**Academic** **Roles**	**Number of Comments**
Announcement: Death of one of dental student at KKU.	News and announcements	Unclassified	121
KSU congratulating a faculty member for her promotion to full professor.			73
PSAU congratulating a faculty member for appointing him as a Vice Dean of Academic Affairs.			66
IAUdent congratulating a faculty member for appointing her as assistant professor.			65
COD at KSU congratulating a faculty member for appointing him as assistant professor.			61

## Discussion

This is the first study to investigate social media use, namely Twitter by dental schools for education, health promotion and professional engagement in Saudi Arabia. The study sheds the light on the possible benefits of Twitter for dental schools. The present study showed that the activity on Twitter for Saudi Arabian dental schools is dynamic with ups and downs, with the highest activity seen in the month of September and the lowest in the month of July. The rise in tweets during the month of September could be associated with the academic activity of dental schools in Saudi Arabia as the academic year starts in September. On the other hand, the month of July coincides with the summer vacation in Saudi Arabia which explains the decline in twitter activity of dental schools. This trend was similar to a study on scholars’ participation in Twitter where tweets where high during academic year and low during summer vacation [16]. In March 2020, there was a sudden increase in dental schools’ Twitter activity compared to other months. This might be related to the COVID-19 pandemic, as more health and academic institutes increased their tweets to spread vital and important information about the pandemic [17]. There was a partial lockdown in Saudi Arabia during the middle of March 2020, which included universities and dental schools. Dental schools like other health organizations used Twitter as a channel of communication with students, employees and patients during that time which may further explain the increased Twitter activity.

In this paper, News and announcements had the highest distribution by 55% among the other main thematic classifications. This confirms that dental schools like other higher education institutes are using Twitter mainly as a platform to share news and updates with students, employees and the community [18,19]. Dental professional communication was the second most tweeted theme especially about dental conferences, workshops, and scientific lectures. Twitter worked as an excellent channel for academic institutes to advertise for these activities [20]. Furthermore, Twitter was used to outline schools’ achievements, raising awareness by educating the community about oral health, and as a general communication channel to celebrate national events [18,19]. Moreover, Twitter and social media in general played a vital role in raising awareness and educating the population about COVID-19 pandemic [21]. Given the effectiveness, efficiency, popularity and reachability of tweets, dental schools should utilize Twitter in delivering health information and health education especially during pandemics.

In this study dental schools exhibited a high interest in community service, as seen by the number of tweets (n = 803, 42%). Most of the tweets were about conferences and workshops as well oral health education which fall under the community service area. In addition, the study covered the period in which COVID-19 pandemic started and almost all dental schools used their accounts to raise awareness about the disease as well as preventive measures. It is worth to mention that community service is an important part of all dental schools’ mission and/or vision and is considered one of the indicators that positively affect the international university ranking [22].

Tweets dissemination and engagement are affected by many tools such as language used and content nature [9,23]. Colorful, high-quality pictures and professionally taken shots have been shown to be an effective strategy in increasing post engagement rate by the nearly 29 times as well as an increase in the number of retweets [24]. On the other hand, tweets containing screenshots receive a significantly lower number of retweets than the ones with professionally taken pictures.

As noticed in the results of this study, several dental schools used different methods to increase followers’ engagement. Improved followers’ engagement can enhance visibility of Twitter accounts and serve as a marketing tool [25]. According to one study on social media engagement in Saudi universities, Twitter was the most frequently used platform to communicate with audiences [26].

To study users’ influence on Twitter, one should consider many factors together including the number of followers, the number of received retweets and likes, the account activity, and the format of used hashtags [27–31]. Considering one factor or variable alone may not be representative of the popularity of an account although there might be a positive correlation between them [27]. For example, Fedushko, 2019 have reported that the use of hashtags resulted in higher popularity by 12.6% and Wadhwa, 2017 reported higher engagement rates up to 3 times more. However, this was not the case, as this did not reflect entirely in our study. This may be attributed to the nature of the tweets and their contents [9]. Twitter accounts branding for dental schools should focus on the perspective of the followers especially students and other important stakeholders. Student loyalty increases when students feel that their dental schools communicate with them well and meets their various needs.

Our study analysis was limited to available tweets posted from July 2019 to July 2020. The data included tweets only from official dental schools’ accounts, which means that tweets from supporting accounts (e.g.: Main university accounts, clubs, or hospital accounts) were excluded. In addition, private dental schools were not included in this study because they did not have a specific account for the dental school. For example, one of the private dental schools Twitter accounts were related to admissions or to the dental services. Also, some schools have well established accounts while others may have recent accounts and therefore would have fewer tweets and followers.

Also, we were limited in the accessibility of some tweets and accounts. It was noted during revision of the data that some of the tweets were not accessible; either because the accounts were either deleted or converted to private accounts, or the tweets were deleted. This blocking may have affected the results because it may show different opinions from the posted public tweets.

Further studies are encouraged to extend the time period of the analysis and include tweets from all dental school related accounts, which is likely to produce more information especially comparing public to private dental schools’ Twitter activity. Moreover, the demographic data of the followers was difficult to determine, this would have helped in further analysis of the tweets. Further studies are needed to understand the roles of the followers in the academic institution (student, faculty, administrator, etc.) and how they interact with the institutions’ accounts.

## Conclusion

This study highlights the importance of Twitter, in dental education especially when it comes to Twitter presence and branding for dental schools. Twitter is a helpful platform to expose dental schools to the community, this can be seen by their academic achievements as well as their active role with community service. The success of dental schools’ twitter accounts depends on the engagement of their followers and attracting new ones. Dental schools are encouraged to choose proper timing for posting tweets. Frequency and type of posts are also important tools for engagement of followers. Never the less, management of Twitter accounts require time, effort and dedicated personnel.

## Supporting information

S1 Data (XLSX)
